# Integrating New Approach Methodologies (NAMs) into Preclinical Regulatory Evaluation of Oncology Drugs

**DOI:** 10.3390/biomimetics10120796

**Published:** 2025-11-24

**Authors:** Maryam Sadat Mirlohi, Tooba Yousefi, Amir Reza Aref, Amir Seyfoori

**Affiliations:** 1 Department of Mechanical Engineering, University of Victoria, Victoria, BC V8W 2Y2, Canada; maryammirlohi@uvic.ca; 2 Department of Clinical Biochemistry, School of Medicine, Shahid Beheshti University of Medical Sciences, Tehran 1985717413, Iran; toobayousefi@sbmu.ac.ir; 3 Mass General Cancer Center, Department of Surgery, Massachusetts General Hospital, Harvard Medical School, Boston, MA 02115, USA; aaref@mgh.harvard.edu; 4 Apricell Biotechnology Inc., Victoria, BC V8W 2Y2, Canada

**Keywords:** NAMs, oncology, in vitro models, drug development

## Abstract

Traditional animal-based preclinical models, including xenografts and genetically engineered mice, have been used for assessing pharmacodynamics, toxicity, efficacy, and safety for decades. Despite their limited ability to mimic human tumor heterogeneity, immune interactions, and microenvironmental complexity, over 90% of oncology candidates that succeed in animal studies fail in clinical trials. The New Approach Methodologies (NAMs), which include patient-derived organoids, organ-on-chip platforms, and AI-driven computational models, provide human-relevant solutions that can improve predictive validity, mechanistic insight, and ethics. Through these technologies, it will be possible to replicate tumor biology specific to patients, to support co-clinical trial designs, and to facilitate biomarker discovery while reducing animal testing. Several recent regulatory reforms, including the Food and Drug Administration (FDA) Modernization Act 2.0 and the European Medicines Agency’s NAM qualification framework, have established clear pathways for the integration of validated NAMs into preclinical drug evaluation. Critically evaluating the scientific rationale, comparative performance, and regulatory context of key NAM platforms in oncology, this review highlights opportunities for synergistic integration, technical refinement, and global harmonization in order to accelerate the development of clinically effective cancer therapeutics based on preclinical findings.

## 1. Introduction

Preclinical oncology has long relied on animal models, xenografts and genetically engineered mice among them, as the default tools for early assessment of pharmacodynamics, toxicity, efficacy, and safety [1]. These systems shaped regulatory expectations and drug-development workflows for decades, and they remain embedded in laboratory practice, contract research organization (CRO) services, and preclinical pipelines [2,3]. Indeed, more than 90% of oncology candidates that show preclinical efficacy in animal studies do not succeed in human trials, a shortcoming driven largely by species-specific differences in pharmacology and disease biology [4]. Yet the persistent failure of many candidates to replicate animal-derived efficacy in humans has profound consequences beyond headline statistics: it increases development timelines, multiplies costs across successive trial phases, and exposes patients and sponsors to avoidable risk. This translational shortfall also drives opportunity cost; promising therapeutic strategies may be deprioritized while resources continue to flow toward compounds that perform well in animals but not people. The cumulative effect is a drug-developed ecosystem that is slower, more expensive, and less efficient at delivering new cancer therapies to patients [5,6].

New Approach Methodologies encompass a broad set of human-relevant, non-animal approaches that span experimental platforms, computational tools, and integrated data strategies [7]. NAMs as an alternative toolkit that can be applied at multiple decision points including early safety screens, candidate prioritization, exploratory toxicology, or as supplemental evidence for regulatory filings. The NAM landscape is heterogeneous in maturity, some approaches, including patient-derived organoids, organ-on-chip microphysiological systems, are already used routinely in industrial screening workflows, others such as mechanistic computational simulations are at the proof-of-concept stage, and they are increasingly incorporated into hybrid testing strategies that blend in vitro, in silico, and human-derived data. Importantly, NAMs are not positioned merely as replacements; in practice they often function as risk-reducing complements that can de-risk clinical translation by providing additional human-relevant evidence earlier in the pipeline [8,9].

Recent regulatory reforms have begun to recognize NAMs as valid sources of nonclinical evidence, but acceptance is conditional. Notably, the FDA Modernization Act 2.0 permits scientifically justified, robust, and fit-for-purpose non-animal methods to support regulatory submissions, and subsequent FDA guidance documents and agency roadmaps have signalled openness to organ-on-chip and computational approaches [10,11]. That requires transparent validation pathways, agreed performance criteria, and community standards for reproducibility and data reporting. Beyond validation, practical adoption faces barriers including the need for standardized protocols, inter-laboratory reproducibility data, commercial scalability, and clear guidance on how NAM-generated endpoints map to regulatory decision criteria. There are also organizational and cultural hurdles, established laboratory practices, legacy regulatory expectations, and intellectual property considerations that slow uptake. Addressing these non-scientific barriers is as important as solving technical ones if NAMs are to meaningfully shift non-clinical evaluation [12,13].

This review aims to situate NAMs within that broader translational and regulatory context and critically evaluate the scientific and regulatory rationale for integrating NAMs into non-clinical oncology frameworks, compare the strengths and limitations of key NAM platforms, and explore strategies for their broader adoption. We will summarize our perspective by offering actionable recommendations for stakeholders, researchers, industry, regulators, and funders on priority areas for standardization, inter-laboratory validation studies, and policy work needed to accelerate responsible NAM integration into oncology pipelines.

## 2. Why Oncology Needs NAMs

### 2.1. Poor Clinical Translation of Animal Models in Cancer

Animal models frequently fail to predict clinical outcomes because the biological context in which a drug is tested differs in important and often unpredictable ways from the human disease setting. Differences in absorption, distribution, metabolism, and excretion (ADME) alter exposure and toxicity profiles underperforming their predictive capabilities of clinical efficacy in oncology, only a small fraction of candidates that show preclinical promise ultimately reach patients [14,15]. These divergences produce two costly errors, including false positives, where compounds that appear efficacious and safe in animals do not translate to humans, and false negatives, where potentially effective therapies are deprioritized after disappointing animal data. This discrepancy not only increases development costs and timelines but also distorts decision-making, as resources may be directed toward candidates unlikely to succeed while more promising options remain underexplored [2]. Although this review primarily emphasizes NAMs and animal models, it is important to acknowledge that standard in vitro 2D monolayers and 3D multicellular cultures often serve as the entry point for mechanistic and drug-response studies due to their low cost, ease of handling, and reproducibility. However, their limited physiological complexity and lack of immune and vascular components constrain their predictive value, which is why they are referenced here as a contextual baseline rather than a central focus of this comparison [16,17].

### 2.2. Tumor Heterogeneity, Resistance, and Immune Dynamics Poorly Modeled In Vivo

Tumors are dynamically evolving ecosystems comprising malignant clones, stromal cells, vasculature, and infiltrating immune populations that evolve under therapeutic pressure. Standard in vivo platforms, such as patient-derived xenografts (PDX) and genetically engineered mouse models (GEMMs), represent only portions of this complexity [18]. PDX models often lose human stromal and immune components and therefore fail to recapitulate the native human immune–tumor crosstalk or the spatial organization that influences drug penetration and immune surveillance. GEMMs can reproduce tumor initiation mechanisms driven by defined oncogenic events but lack the mutational and microenvironmental diversity found in human cancers [19,20]. Both approaches struggle to model temporal processes such as adaptive resistance, treatment-induced phenotypic switching, hypoxia-driven selection, and the spatially resolved interactions between tumor and immune cells that frequently determine response to immunotherapies and combination regimens. These specific shortcomings limit the utility of standard animal models for evaluating next-generation immuno-therapies, adaptive dosing strategies, and tactics designed to prevent or overcome resistance [21].

Collectively, the divergence between murine and human pharmacology leads to efficacy overestimation and toxicity underprediction, causally contributing to the >90% attrition rate observed when transitioning from animal studies to clinical trials. NAMs aim to mitigate these translational gaps by more accurately recapitulating human-specific metabolic and immune dynamics [7,22]. To provide a structured overview, Table 1 summarizes key comparative features of conventional in vitro and animal-based models versus NAMs across translational, technical, and regulatory dimensions.

### 2.3. NAMs as Tools for Improving Relevance, Efficiency, and Patient-Centric Evaluation

NAM platforms are designed to address mechanistic blind spots inherent to in vivo models. Patient-derived organoids retain patient-specific clonal architecture and can be propagated to test panels of drugs against intrinsic and acquired resistance phenotypes, making them useful for ex vivo sensitivity profiling and biomarker discovery [25,26]. A cancer-on-chip (CoC) systems introduce physiologically relevant flow, endothelial barriers, and multicellular niches, enabling experiments that probe drug delivery, transendothelial migration, and immune cell trafficking under controlled shear and gradient conditions [27]. 3D bioprinting offers tailored control over extracellular matrix composition and tissue geometry to investigate how mechanical and spatial cues influence invasion and drug responsiveness. Complementary computational and mechanistic models synthesize longitudinal, single-cell and multi-omic readouts from these platforms to infer trajectories of clonal evolution and to prioritize combination strategies predicted to delay resistance [23]. Importantly, NAMs are best viewed as part of an integrated strategy so that they can increase throughput and mechanistic resolution in early screening and inform more focused in vivo tests or clinical trial designs, while acknowledging current limitations such as whole-body pharmacokinetics and endocrine interactions remain outside the scope of isolated in vitro systems. Used together, these tools enable more patient-centric, hypothesis-driven decision making and can improve the alignment between preclinical evidence and the clinical questions faced in trial design [28,29].

Although this review focuses primarily on animal-based and next-generation NAM platforms, it is important to acknowledge the pivotal role of conventional in vitro systems, including 2D monolayers and multicellular 3D cultures, as the first tier of preclinical evaluation. These models offer cost-effective and reproducible environments for mechanistic studies and early-stage drug screening; however, their lack of immune, vascular, and systemic complexity limit translational predictivity [30,31]. For this reason, they are presented here as an essential baseline within the comparative framework but were not the central focus of this review, which emphasizes human-relevant alternatives under current regulatory transformation

## 3. Comparative Overview of Key NAM Platforms

**Figure 1:**
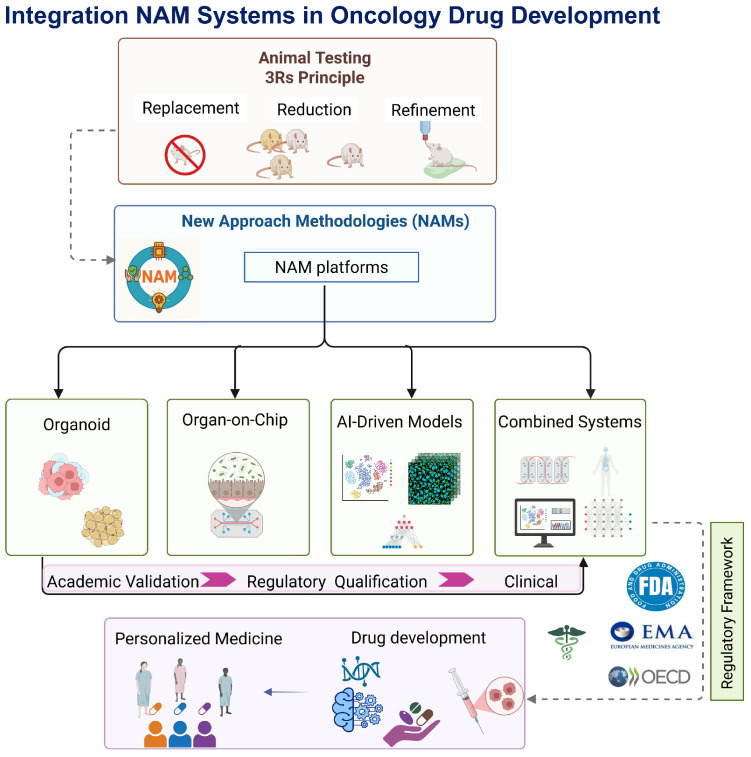
Integration of New Approach Methodologies (NAMs) in oncology drug development. NAM platforms, including organoids, organ-on-chip, AI-driven models, and combined systems, support personalized medicine and drug development while reducing reliance on animal testing (3Rs principle) and aligning with regulatory frameworks (FDA, EMA, OECD). Created with BioRender.com (https://BioRender.com/jiaoyxe (accessed on 14 September 2025)).

The landscape of New Approach Methodologies for oncology is marked by a cluster of new platforms that attempt to optimize the predictive value of preclinical models with minimal reliance on animal testing. Among them, organoids, organ-on-chip (OoC) systems, artificial intelligence (AI) enabled models, and their hybrid variants are the most developed and rapidly evolving approaches [32]. These platforms and their integration in oncology drug development are summarized in Figure 1.

### 3.1. Organoids

Organoids are three-dimensional cell aggregates derived from patient tumors or pluripotent stem cells that retain most of the histological, genetic, and phenotypic characteristics of the source tissue. Their capacity to preserve intratumoral heterogeneity makes them valuable for personalized drug testing, functional genomics, and biomarker discovery [33]. The establishment of patient-derived organoid biobanks has enabled parallel testing of therapeutic candidates across genomically diverse samples, improving the translational relevance of preclinical findings [34]. Organoid models do have important limitations including a lack of functional vasculature and systemic immune components, which constrains their ability to model drug delivery, tumor–immune interactions, and metastatic dissemination [25]. In addition, batch-to-batch variability, often driven by differences in extracellular matrix composition or culture conditions, raises reproducibility concerns for large-scale studies [35]. Recent literature highlights both progress and outstanding challenges. Hu et al. review advances in using patient-derived organoids (PDOs) for hepatobiliary and pancreatic cancers, noting improvements from co-culture and microfluidic approaches that better recapitulate tumor environments; they emphasize the strong predictive potential of PDOs while calling for integration of genomics, engineering, and AI to enhance clinical applicability [36]. Bhattacharya et al. describe scaffold-free 3D breast cancer constructs that incorporate multiple cell types (fibroblasts, HUVECs, cancer stem cells) and cellular manipulation strategies to reproduce tumor heterogeneity and microenvironment complexity; these models better reflect in vivo behaviors such as metastasis, angiogenesis, and drug resistance, offering a physiologically relevant platform for therapeutic screening [37]. In addition, a study by Lee et al. developed a low-coherence holotomography (HT) technique for long-term, label-free, high-resolution 3D imaging of live mouse small intestinal organoids. It enabled detailed visualization of morphological features and dynamic cellular activities at subcellular resolution, facilitating quantitative analysis of organoid growth, viability, and response to drugs. It also provides a comprehensive platform for real-time monitoring of organoid development and pharmacological effects [38]. For example, patient-derived colorectal organoids have predicted individual clinical responses to cetuximab with >80% concordance to patient outcomes [39]. Together, these developments strengthen the translational promise of organoid-based models while underscoring the need for continued standardization and technological integration to fully realize their potential in precision oncology.

### 3.2. Organ-on-Chip

Organ-on-chip systems mitigate several of the translational limitations of organoids by merging microfluidics with tissue engineering to recreate dynamic physiological conditions. These platforms afford precise control over variables such as shear stress, nutrient gradients, and mechanical cues, parameters that are crucial for modeling in vivo tumor physiology [40]. In oncology, OoC models can reproduce vascular interfaces, metastatic invasion routes, and immune cell infiltration, offering a more nuanced readout of tumor behavior under therapeutic pressure. [41,42]. Their ability to integrate multiple cell types (endothelial, immune, stromal) permits reconstruction of microenvironmental interactions that static cultures cannot capture [24,43]. Nonetheless, device complexity, high fabrication costs, and limited throughput currently constrain their scalability for large-scale drug screening. Ongoing efforts to standardize device architectures and automate workflows aim to address these barriers [44]. In this context, Liu et al. examines the state and future prospects of CoC technologies for precision oncology, highlighting advances that incorporate tumor heterogeneity, vasculogenesis, and patient-derived material to increase physiological relevance. They argue that combining CoC platforms with embedded sensors, artificial intelligence, and standardized methods could improve throughput and reproducibility, helping to bridge the translational gap between preclinical models and clinical outcomes. They conclude that next-generation CoC models, augmented with sensors, AI, and standardization efforts, hold promise to bridge the gap between preclinical studies and clinical outcomes [27]. A breast-cancer-on-chip co-culture model successfully reproduced trastuzumab resistance mechanisms and accurately predicted therapeutic response validated in xenograft studies [41,45]. Ultimately, these innovations aspire to enable more predictive, personalized cancer therapies and to accelerate translational research

### 3.3. AI-Driven Models

AI-driven approaches serve as a computational complement to biological NAMs. Using machine-learning and deep-learning techniques, AI can fuse high-dimensional datasets, ranging from imaging and multi-omics to pharmacokinetic and toxicological readouts, to build predictive models of safety and therapeutic response [46]. In oncology, such models are increasingly applied to patient stratification, discovery of resistance mechanisms, and optimization of combination regimens [47]. AI can also extract actionable signals from NAM outputs (for example, time-lapse organoid imaging or sensor streams from OoC platforms), revealing non-obvious correlations and candidate biomarkers. Nevertheless, the reliability of AI predictions is highly dependent on data quality, diversity, and representativeness [48]. Algorithmic transparency and interpretability remain critical concerns, particularly in the regulatory context, where explainability is essential for decision-making [49]. Maramraju et al. discuss how AI is advancing analyses of hPSC-derived organoids, improving quality control, enabling label-free identification, and enhancing 3D image reconstruction from complex organoid datasets. The article highlights AI’s potential to enhance the quality control of organoid fabrication, enable label-free organoid recognition, and improve three-dimensional image reconstruction of complex organoid structures [50]. Recent advances demonstrate that artificial intelligence enables automated, high-speed phenotypic analysis and drug response profiling of cancer organoids by integrating deep learning pipelines for segmentation, morphology quantification, and predictive modeling [48]. AI pipelines that integrate organoid morphology and multi-omic profiles have demonstrated strong predictive performance for drug sensitivity, supporting the feasibility of multimodal prediction frameworks [51,52]. A central challenge in AI–organoid work is that experimental variability and inconsistent dataset curation can undermine model generalizability and limit confidence in AI-driven inferences.

### 3.4. Combined Systems (AI + Biological NAMs)

The integration of AI with biological NAMs offers a promising avenue for achieving a more comprehensive, mechanistically grounded, and patient-relevant preclinical framework. In this conjoin strategy, biological platforms such as organoids and OoC devices generate profound, physiologically relevant datasets, which are analyzed by AI systems to guide experimental design, predict clinical responses, and identify best therapeutic regimens [53,54]. This digital-biological synergy could dramatically accelerate oncology drug pipelines, reduce attrition rates, and potentially replace certain categories of animal testing altogether [55]. However, for such hybrid systems to gain widespread adoption, regulatory frameworks must evolve to recognize and validate integrated datasets. Initiatives such as the FDA Modernization Act 2.0, ARPA-H’s CATALYST program, and NIH’s ARIVA (National Institutes of Health/Accelerating Research for Innovative and Validated Alternatives) Office indicate a growing institutional commitment to fostering these innovations, but consistent standards for validation, interoperability, and data governance will be essential [12,56].

In summary, organoids, organ-on-chip systems, AI-driven models, and their combinations each contribute unique strengths to the oncology research toolkit. While organoids excel at modeling tumor heterogeneity, OoC devices capture dynamic physiological processes, and AI enables large-scale integrative predictions, their convergence holds the greatest promise for a future of preclinical testing that is not only more predictive but also more ethical and efficient. The success of this transformation will depend on overcoming technical limitations, improving reproducibility, and establishing robust, internationally harmonized regulatory pathways. A concise summary of the major NAM platforms, their advantages, and limitations is presented in Table 2 to facilitate cross-platform comparison.

## 4. Regulatory and Policy Context

### 4.1. Recent Shifts: FDA Modernization Act, NIH’s ARIVA, EMA Initiatives

Recent years have seen regulatory agencies increasingly recognize the limitations of traditional animal-based models for predicting human outcomes, particularly in oncology, and have begun to encourage the use of NAMs to assess nonclinical outcomes [7]. In 2022, the FDA Modernization Act 2.0 marked a major milestone by removing the statutory requirement for animal testing in nonclinical drug development when scientifically justified and validated alternatives are available [11]. This shift has been put into practice through the FDA’s 2025 Roadmap to Reducing Animal Testing, which identifies priority areas for NAM integration, including the use of organ-on-chip platforms and in silico modeling as primary evidence, especially for biologics and monoclonal antibody development [57]. Within the NIH, parallel efforts have advanced the adoption of human-relevant research models across federally funded programs, including initiatives to restrict funding for animal-only studies in specific contexts and to speed up the qualification of advanced in vitro systems. Internationally, the European Medicines Agency (EMA) and the Organization for Economic Co-operation and Development (OECD) have established policies to facilitate NAM adoption through early scientific advice, voluntary qualification pathways, and inclusion of NAM-based evidence in marketing authorization dossiers. In addition to aligning regulatory innovation with the 3Rs principle, these reforms demonstrate a growing convergence of regulatory initiatives around robust, human-relevant models [58,59]. International regulatory frameworks for NAMs are increasingly interconnected through formal harmonization mechanisms. While the FDA and EMA operate within their respective regulatory jurisdictions, they align their validation principles via OECD guidance and ICATM coordination. ICCVAM provides the U.S. reference framework for NAM validation, which in turn integrates with the OECD Mutual Acceptance of Data (MAD) system to enable globally recognized validation outcomes. This collaborative structure allows a single NAM validation study to support multiple regulatory submissions, reducing duplication, accelerating decision-making, and streamlining oncology drug development [7,60,61].

### 4.2. Oncology-Specific Flexibility

There is increasing recognition that alternative models-including in Silico, ChemoCo, and Advanced in Vitro Systems-can improve safety predictions for humans and modernize regulatory decision making, serving as a viable complement or replacement for traditional animal-based approaches [62]. Oncology approval pathways are increasingly allowing early market access for targeted therapies by accepting early-phase or surrogate endpoint data, along with post-market confirmation requirements. The ability of NAMs to generate human-relevant mechanistic evidence with organoids, organ-on-chip systems, or computational models may enhance the scientific basis for conditional approvals. NAMs can be validated and applied in well-defined contexts of use, improving translational reliability and aligning with global trends toward ethical, patient-centered drug development [63]. In drug research and development, artificial intelligence converges with advanced human specific platforms, such as organoids and organ chips. Using this approach, it is possible to identify promising drug candidates with greater predictive accuracy and efficiency, thus reducing the need for animal testing and accelerating discovery [64]. In several instances, well-characterized biologics have advanced to early-phase clinical trials based on robust human-relevant data, with minimal or no animal testing. These cases highlight oncology as a testing ground for NAM-driven regulatory innovation and show that regulators can accept validated alternatives in place of animal studies when justified. Recent cases illustrate this regulatory evolution. For instance, a bispecific anti-PD-1/CTLA-4 antibody candidate advanced to a first-in-human trial after non-clinical safety profiling in human-immune-cell-on-chip assays and humanized ex vivo cultures, supported by FDA pre-IND consultation. Similarly, CAR-T constructs optimized through organoid co-culture assays have entered Phase I evaluation without additional murine efficacy studies, reflecting growing regulatory confidence in NAM-derived mechanistic evidence [65,66].

Beyond non-animal alternatives, comparative oncology provides a valuable translational bridge. These models share genomic and microenvironmental features with human tumors and can refine drug development by generating clinically relevant pharmacokinetic and safety data without increasing rodent use. While not NAMs per se, they align with the 3Rs by reducing reliance on traditional laboratory species and supporting ethically grounded translational research [67,68,69].

### 4.3. Challenges: Lack of Harmonized Criteria, Context-of-Use Validation Still Evolving

Context of Use (CoU) refers to the conditions under which a NAM is intended to support the development and regulatory evaluation of human or veterinary medicines. It is beneficial to establish the CoU at an early stage of NAM development so regulatory bodies, such as the EMA, can provide focused scientific advice. The EMA’s qualification process requires developers to specify how the NAM will be used, whether as a replacement for existing regulatory requirements or as part of an integrated weight-of-evidence approach combining pharmacology, pharmacokinetics, in vitro, and in vivo data [70].

**Figure 2:**
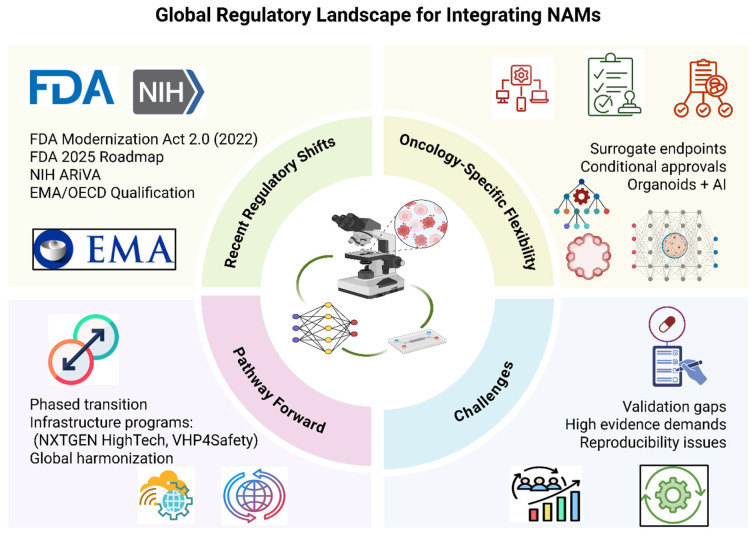
Regulatory and policy context for integrating NAMs in oncology drug development, emphasizing recent regulatory shifts, oncology-specific flexibility, key challenges, and future harmonization efforts. Created with BioRender.com (https://BioRender.com/i6obo02 (accessed on 14 September 2025)).

In the March 2024 report from the Interagency Coordinating Committee on the Validation of Alternative Methods (ICCVAM) Validation Workgroup report, 17 U.S. federal agencies, such as the FDA, NIH, and EPA, propose a modernized, context-of-use-specific validation framework for NAMs. The performance criteria are tailored to the intended regulatory application, focusing on biological relevance, mechanistic clarity, reproducibility, and robust data integrity over rigid, one-size-fits-all standards. As well as promoting regulatory and international harmonization, this framework promotes initiatives from the Organization for Economic Co-operation and Development (OECD), International Cooperation on Alternative Test Methods (ICATM), and the International Council for Harmonization of Technical Requirements for Pharmaceuticals for Human Use (ICH). The use of organoids, organ-on-chip systems, and AI-driven models in both efficacy and safety evaluations could be speeded by such a flexible approach in oncology, where failure rates are high and tumor biology is complicated [71]. See Figure 2 for a schematic overview of regulatory pathways and NAM integration in oncology drug development.

In Europe, initiatives such as the Netherlands’ RIVM (Rijksinstituut voor Volksgezondheid en Milieu/National Institute for Public Health and the Environment, The Netherlands) roadmap emphasize reproducibility, stakeholder engagement, and regulatory harmonization as a framework for integrating NAMs into pharmaceutical safety assessment. In this roadmap, a phased transition from exploratory use to regulatory acceptance is presented, supported by coordinated infrastructure programs such as NXTGEN HIGHTECH, which is developing advanced organ-on-chip platforms, and Virtual Human Platform for Safety Assessment (VHP4Safety), which simulates the safety of humans virtually. Oncology drug evaluation aims to strengthen predictive validity, reduce reliance on animal models, and align with broader EU strategies to promote human-relevant, ethically responsible drug development [72].

EMA reports that fragmented scientific advice in the EU leads to duplication, inconsistent guidance, and delays—issues especially problematic for newly developed methods such as NAMs. As part of the proposed EU pharmaceutical legislation, the EMA, national regulators, and Health Technology Assessment (HTA) bodies will be able to provide clearer, harmonized advice and reduce procedural redundancy in the process. Oncology could benefit from such reforms by incorporating human-relevant models-such as organoids, organ-on-chip systems, and AI-driven simulations, into nonclinical evaluation, while maintaining rigorous evidentiary standards and speeding up patient access to innovative treatments [73].

### 4.4. Implementation Challenges for NAM Integration

Integrating NAMs is hindered by fragmented validation standards, limited regulatory confidence, and unclear legal pathways. There are often higher evidentiary demands for NAM data than for traditional animal studies due to variability in assay reproducibility and regulatory expectations. In oncology, many NAM platforms are also unprepared for complex endpoints such as chronic toxicity and multifactorial disease modeling. To address these issues, the FDA Modernization Act 2.0 and OECD guidelines advocate harmonized validation frameworks, robust interlaboratory studies, and clearer regulatory alignments [74].

In addition to policy and regulatory advances, methodological studies have highlighted critical considerations for integrating NAM-derived data into safety and efficacy assessments. For example, Carlson et al. evaluated the application of high-throughput, thyroid-relevant in vitro assays for pesticide chemicals, using in vitro-to-in vivo extrapolation (IVIVE) to derive equivalent administered doses (EADs). Their findings demonstrated that model parameterization substantially influenced EAD estimates, in some cases producing values lower or higher than traditional animal-based points of departure (PODs). This variability underscores the importance of rigorous parameter selection, transparent modeling assumptions, and incorporation of uncertainty ranges when NAM outputs are intended for regulatory decision-making. Such analyses not only illustrate NAMs’ potential for generating human-relevant toxicological insights but also emphasize the need for robust validation and sensitivity testing to ensure reproducibility and context-of-use reliability in oncology drug development frameworks [75].

Although the focus of this review is replacement through NAMs, refinement of existing in vivo models remains a vital parallel track within the 3Rs framework [76,77]. Advances in humanized mouse strains, spontaneous disease models, and improved welfare protocols are enhancing physiological and immunological fidelity while reducing animal use [78]. Replacement and refinement should be regarded as synergistic efforts that collectively strengthen translational accuracy and ethical responsibility.

### 4.5. Validation Frameworks and Fit-for-Purpose Qualification of NAMs

NAMs are context-specific and aim to demonstrate that a given method is reliable, reproducible, and suitable for a defined regulatory application [32,71]. Regulatory bodies such as ICCVAM, OECD, and EMA recommend a tiered approach encompassing:

(i) Scientific benchmarking, comparison of NAM outputs against established “gold-standard” in vivo or clinical reference data for efficacy, toxicity, or pharmacokinetics [79].
(ii) Inter-laboratory reproducibility testing, multi-site ring studies following OECD GD 211 and ICCVAM templates to verify robustness across settings.
(iii) Performance metrics, quantitative evaluation of reproducibility, sensitivity, specificity, and concordance with reference methods, with acceptance thresholds typically >80% agreement for regulatory qualification [80].
(iv) Clinical correlation, mapping NAM-derived endpoints to early-phase human trial biomarkers to confirm translational validity.

Fit-for-purpose validation, therefore, emphasizes biological relevance and decision-context suitability rather than universal criteria, enabling flexible qualification of NAMs across diverse oncology applications [71].

Table 3 outlines the key elements of NAM validation, including benchmarking, reproducibility, performance metrics, and clinical correlation, along with practical examples and relevant regulatory guidance.

Notably, the Emulate Liver-Chip was accepted by the U.S. FDA’s Center for Drug Evaluation and Research (CDER) as part of a drug-induced liver-injury assessment (2023), demonstrating >87% sensitivity and >90% specificity relative to clinical hepatotoxicity data (FDA CDER Report 2023). Similarly, cardiac microtissue platforms have been qualified under EMA’s Innovation Task Force for early cardiotoxicity screening (EMA 2024). These precedents illustrate the quantitative reliability thresholds currently guiding NAM qualifications.

## 5. Future Outlook

NAMs, which include in vitro assays, in silico modeling, omics-based profiling, and organ-on-a-chip platforms, are poised to transform nonclinical regulatory evaluation in oncology drug development. In addition to enabling faster decision-making, increasing human biological relevance, and reducing costs, these approaches offer a viable alternative to animal studies. However, regulatory agencies, including the U.S., continue to encounter limitations, particularly in modeling certain human biological endpoints. In addition to the FDA, EMA has made clear strategic commitments to the qualification and implementation of validated NAMs. It is expected that international efforts in method validation and regulatory harmonization will further accelerate their acceptance. The oncology field offers a unique opportunity for rapid integration of NAM. A growing need for novel cancer therapeutics, the wide availability of molecular and clinical datasets, and the use of advanced translational models such as patient-derived organoids, circulating tumor DNA (ctDNA) monitoring, and digital pathology all contribute to a fertile environment for the deployment of NAMs. The regulatory experience in oncology, specifically with monoclonal antibodies and targeted agents, has already shown that the use of animal models is insufficient to generate early and more patient-relevant safety and efficacy insights.

Building on earlier discussions of comparative model performance and evolving regulatory frameworks, the future trajectory of oncology drug development is expected to shift toward integrated, human-relevant pipelines. The predictive advantages of organoid and organ-on-chip platforms, together with increasingly structured validation pathways, provide a strong foundation for the mainstream regulatory adoption of NAMs within the next decade. To maximize the value of NAMs in oncology, sustained interdisciplinary collaboration is essential. It is critical that molecular biologists, computational modelers, clinicians, and regulators work together to define scientifically robust and regulatory-acceptable endpoints. Incorporating NAM data into nonclinical packages with greater confidence can be achieved through early and proactive engagement with regulatory bodies. A global harmonization of technical and validation standards will be necessary to ensure that NAM-generated evidence is accepted across multiple jurisdictions, thereby streamlining the drug development process. Introducing AI is a powerful accelerator of this transformation. In addition to in silico toxicity prediction and pharmacokinetic-pharmacodynamic (PK-PD) modeling, AI-enabled tools improve NAM predictive value by integrating multi-omics and clinical trial datasets. A number of regulatory agencies are beginning to acknowledge the role of AI by recommending that algorithm design, training datasets, and maintenance procedures be transparently disclosed in submissions where AI has influenced safety or efficacy conclusions. Using AI-driven bio simulation platforms, oncology drug sponsors could design and execute preclinical development virtually, reducing or even eliminating the need for animal testing. Regulatory science is advancing toward a future where AI-supported, animal-free submissions are an achievable standard in this evolving landscape. It paves the way for more accurate, efficient, and ethically responsible drug development, allowing broader application across therapeutic areas.

## 6. Conclusions

A variety of NAMs, including patient-derived organoids, organ-on-chip systems, and AI-driven models, provide robust, human-relevant alternatives to traditional animal-based oncology models. The ability to reproduce tumor complexity more accurately can lead to improved translational predictability, more customized strategies, and a reduction in attrition in clinical trials. A number of regulatory advances are paving the way for their adoption, including the FDA Modernization Act 2.0 and the EMA qualification framework. It will be essential to overcome challenges in standardization, reproducibility, and context-of-use validation. NAM integration could lead to faster, more accurate, and ethically aligned oncology drug development, ultimately leading to safer and more effective cancer therapies.

## Figures and Tables

**Table 1:** Comparative overview of traditional preclinical models and New Approach Methodologies (NAMs) in oncology drug development.

Feature	Conventional 2D/3D Cell Cultures	Animal-Based Models (Xenografts, GEMMs)	New Approach Methodologies (NAMs)
Biological relevance to humans	Limited; simplified monolayers lack systemic and microenvironmental context.	Moderate; capture systemic interactions but differ genetically and immunologically.	High; built on human-derived tissues or computational data.
Tumor heterogeneity	Very low; clonal and homogeneous.	Partial; species-specific tumor evolution.	High; patient-derived organoids and CoC systems retain clonal diversity.
Immune–tumor interactions	Absent.	Species-specific and incomplete.	Human immune components can be incorporated.
Microenvironment complexity	Minimal; lacks ECM architecture and mechanical cues.	Moderate; stromal and vascular components present but non-human.	Advanced; recreate flow, shear stress, and ECM via microfluidics or bioprinting.
Throughput and scalability	High; inexpensive and suitable for screening.	Low; costly and time-consuming.	Moderate; platform-dependent.
Ethical considerations	Non-animal; ethically acceptable.	Involves animal use.	Fully aligned with 3Rs principle.
Regulatory acceptance	Informal use for early discovery; limited for safety evaluation.	Historically accepted standard.	Increasing acceptance through FDA Modernization Act 2.0 and EMA frameworks.
Cost and time efficiency	Very low cost; rapid.	High cost; long development.	Moderate; initial investment, then lower cost per assay.
Reproducibility	High under standardized conditions.	Variable; inter-animal and inter-lab differences.	Improving with standardization; still evolving.
Translational predictability	Low; oversimplified systems.	Limited; ~90% failure in translation.	High; human-relevant and mechanistic.
Ref	[14,23]	[4,5,6]	[7,8,10,24]

**Table 2:** Summary of major NAMs platforms and their principal advantages and limitations in oncology research.

NAM Platform	Description	Advantages	Limitations	Ref
Advanced 2D/3D Human Cell Systems (Pre-NAM bridge)	Human cell lines or spheroids are used for preliminary mechanistic and toxicity screens.	Low-cost, high-throughput entry point; useful for initial pharmacological profiling.	Lack systemic integration; limited immune and vascular mimicry.	[14,17]
Patient-Derived Organoids (PDOs)	3D cultures derived from patient tumors retaining histological and genetic fidelity.	Preserve intratumoral heterogeneity; suitable for drug sensitivity testing, functional genomics, and biomarker discovery.	Lack vasculature and systemic immune components; batch-to-batch variability due to matrix or culture conditions.	[25,34]
Organ-on-Chip (OoC)/Cancer-on-Chip (CoC)	Microfluidic devices mimicking physiological microenvironments and tissue-tissue interfaces.	Simulate vascular flow, mechanical stress, and immune infiltration; improve prediction of pharmacokinetic and immunotherapy responses.	Technically complex; limited scalability; requires standardization and automation for widespread use.	[40,41,43]
3D Bioprinting Models	Biofabrication of tumor tissues using hydrogels and patient-derived cells.	High spatial control; replicate matrix stiffness, geometry, and tumor microenvironment for mechanistic and invasion studies.	Limited reproducibility across laboratories; still under validation for regulatory acceptance.	[28,29]
AI-Driven Computational Models	Machine-learning algorithms integrating omics, imaging, and pharmacological datasets.	Predict efficacy/toxicity; support in silico clinical trials; accelerate target identification.	Depending on dataset diversity, quality, and explainability, interpretability challenges for regulators.	[46,47,49]
Hybrid Systems (AI + Biological NAMs)	Integration of computational and experimental NAMs to enhance translational predictability.	Combine mechanistic insights with predictive analytics; accelerate drug prioritization; reduce reliance on animal models.	Require harmonized data governance, algorithmic transparency, and validation frameworks.	[53,54,56]

**Table 3:** Key components of NAM validation pathways for regulatory acceptance.

Validation Element	Description	Practical Example	Regulatory Alignment	Ref
Context-of-use definition	Defines NAM purpose (replacement, supplement, refinement) and target decision context	OoC for vascular permeability	EMA qualification advice; FDA context-of-use guidance	[81]
Benchmarking	Quantitative comparison of NAM outputs against gold-standard animal or clinical endpoints	OoC toxicity vs. animal LD50 and clinical data	ICCVAM, OECD validation principles	[71]
Inter-laboratory reproducibility	Replication of results across sites with standardized protocols	Multi-site PDO drug-response panel	OECD validation templates; ICCVAM study designs	[82]
Performance metrics	Sensitivity, specificity, reproducibility, uncertainty analysis, predictive value	ROC/AUC comparing NAM vs. clinical response	OECD, ICCVAM, FDA expectations	[83]
Clinical correlation	Alignment of NAM outputs with early-phase clinical trial data	PDO response concordance with patient outcomes	FDA/EMA scientific advice and qualification	[81]

## Abbreviations

3Rs (Replacement, Reduction, and Refinement), AI (Artificial Intelligence), ARIVA (Accelerating Research for Innovative and Validated Alternatives), CAR-T (Chimeric Antigen Receptor T cell), CoC (Cancer-on-a-Chip), CoU (Context of Use), ctDNA (Circulating Tumor DNA), EAD (Equivalent Administered Dose), EGFR (Epidermal Growth Factor Receptor), EMA (European Medicines Agency), FDA (Food and Drug Administration), GEMM (Genetically Engineered Mouse Model), hPSC (Human Pluripotent Stem Cell), HT (Holotomography), HTA (Health Technology Assessment), ICATM (International Cooperation on Alternative Test Methods), ICCVAM (Interagency Coordinating Committee on the Validation of Alternative Methods), ICH (International Council for Harmonisation of Technical Requirements for Pharmaceuticals for Human Use), IVIVE (In Vitro to In Vivo Extrapolation), NAM (New Approach Methodology), NOC/c (Notice of Compliance with Conditions), OECD (Organisation for Economic Co-operation and Development), OoC (Organ-on-Chip), PDX (Patient-Derived Xenograft), PDO (Patient-Derived Organoid), PK-PD (Pharmacokinetic-Pharmacodynamic), POD (Point of Departure), RIVM (Rijksinstituut voor Volksgezondheid en Milieu), VHP4Safety (Virtual Human Platform for Safety Assessment).
